# Synthesis and Characterization of Fe_3_O_4_/Chitosan/TiO_2_ Nanoparticle Novel Nanocarrier and Investigation of In Vitro Release of Vancomycin on HeLa Cancer Cell

**DOI:** 10.1155/ijbm/6164871

**Published:** 2025-05-04

**Authors:** Elahe Mohamadi Bian, Ayoub Moghadam

**Affiliations:** Department of Materials Science and Engineering, Razi University, Kermanshah, Iran

**Keywords:** anticancer, chitosan, HeLa cells, magnetite nanoparticles, TiO_2_ nanoparticles, vancomycin

## Abstract

Nanocarrier systems have gained significant attention in recent decades as an alternative to conventional drug delivery methods, which often suffer from various limitations. In this study, Fe_3_O_4_/chitosan/TiO_2_ nanoparticles were synthesized as a novel nanocarrier for targeted drug delivery. The loading efficiency and controlled release behavior of vancomycin from the nanocarrier were evaluated under in vitro conditions using HeLa cancer cells. The in vitro study of the drug release behavior showed that the implementation of a titania coating significantly diminishes the drug release rate. Specifically, approximately 90 ± 0.2% of the drug is released over a period of 16 h for samples without the titania coating, while samples with the coating exhibit a release time of 25 h. The MTT assay indicates that the application of TiO_2_ nanoparticles on the nanocarrier resulted in a decrease in cell viability from 90 ± 3% to 50 ± 2% at concentrations of 100 μg/mL and 500 μg/mL, respectively. These findings highlight the potential of the Fe_3_O_4_/chitosan/TiO_2_ nanocarrier as an efficient system for controlled and targeted drug delivery applications.

## 1. Introduction

Today, the most common way to use drug is by mouth and injection. These methods at first introduce a very large level of the drug into the blood, but its effective amount decreases greatly during the treatment after a short period of time. This effect is known as the peak and valley effect and can cause serious side effects to the patient. First of all, the concentration of the drug after its administration can reach a toxic and dangerous level. After that, the concentration of the drug reaches below the therapeutic window, which can make the entire treatment process ineffective and inconclusive. The next problem with these types of traditional methods for drug release is that many commonly used antibiotics and drug molecules may become inactive in the digestive system (liver, kidney, and stomach). Precisely for this reason, the effect of the drug release on the target organ will be very weak. Therefore, the drugs should be administered through intramuscular or intravenous injection, both of which will be inconvenient for patients [1, 2].

To overcome these problems and difficulties, new selective and more effective localized release systems will be needed. New achievements in biomedical sciences along with the development of new materials and technologies have led to tremendous advances in the field of drug release control systems. The main goal of controlled drug release is to provide a precise and specific amount of drug in a place, especially inside the body, and then targeted release of it to reach maximum of therapeutic effects [3–5]. In recent decades, nanotechnology has significantly advanced across various medical fields, particularly in targeted drug delivery systems [6]. This approach involves preparing drugs for transport to damaged tissues by either loading them onto the surface of nanomaterials or encapsulating them within their structure [7]. Extensive research has explored the use of nanoparticles as carriers for drugs and bioactive molecules, aiming to enhance therapeutic efficacy, improve drug administration, and minimize side effects [6, 8]. One of the reasons for the importance of nanoparticles in drug delivery is to deliver a large amount of medicinal substance to the target and damaged tissue, due to the very high ratio of surface area to volume in nanoparticles, as well as the possibility of directing and controlling the release of drugs [9]. In recent years, the use of smart release systems has attracted the attention of many researchers in this field. In these systems, by using some special properties and applying external stimuli, including physical (thermal, magnetic, electrical, optical, mechanical, and ultrasound), chemical (stimulation by pH and oxidation–reduction reactions), and biological (different biomolecules and enzymes), the drug is directed to the desired area with a certain amount [10]. Among these methods, release systems based on nanoparticles that have the ability to be controlled through a magnetic field have been given a lot of attention. They, by using a magnetic field, can deliver a greater proportion of the initial amount of the drug to a specific location, do not affect other biological tissues, and reduce the harms of the drug [11, 12].

**Figure 1:**
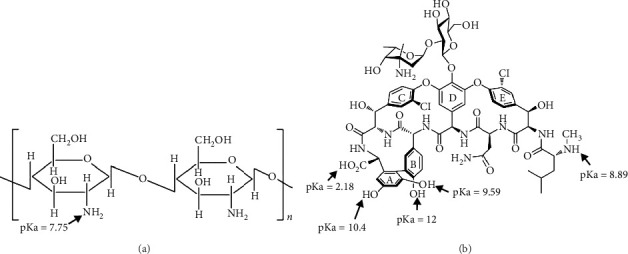
(a) Chemical structure of chitosan polymer and (b) the structure of vancomycin drug.

Nanoparticles with ferromagnetic properties with the general formula MFe_2_O_4_ (where M can be Fe, Co, Ni, Zn, and Mn metals) are good candidates for medical applications [13]. Among these nanoparticles, superparamagnetic magnetite has been considered more than other nanoparticles as drug carriers due to their low toxicity, degradability and biocompatibility, suitable dispersion in aqueous environments, and high reactive surface area [14–17]. The superparamagnetic behavior of these nanoparticles is that they exhibit strong magnetic behavior in the presence of an external magnetic field, but after removing the field, there is no residual magnetic force between the particles, which leads to easy dispersion of the particles and prevents particles from agglomerating [18]. Due to the variety of structures and different applications of iron oxide nanoparticles, several methods can be used for the synthesis of these nanoparticles. The choice of the synthesis method depends on the type of particles needed and their application, and the choice of a suitable synthesis method is directly related to the characteristics and properties of the nanoparticles including shape, particle size distribution, surface chemistry, and their magnetic properties [19]. The most important chemical methods are used to include co-precipitation, microemulsion, thermal decomposition, sol–gel, hydrothermal process, and condensation from a vapor phase. However, the co-precipitation method is the most common method for synthesizing iron oxide nanopowders [20]. To create a stable aqueous dispersion of magnetic nanoparticles for biomedical applications, a ligand exchange step is required. These ligands can also facilitate the fusion of nanoparticles with target biomolecules, drugs, or active polymers. Coating the surface of magnetic nanoparticles with biopolymers can lead to easier separation of the drug and better biocompatibility of nanoparticles, as well as the possibility of using chemicals to connect nanoparticles to the target component [21–23]. Among the polymers that have been used as coatings so far, chitosan is a polymer with high biocompatibility for targeted drug delivery. Chitosan is a biopolymer with high adhesion, biocompatible, biodegradable, hydrophilic, and non-toxic with a positive charge (Figure 1(a)). The presence of amine functional groups on its surface creates a special property that makes chitosan very useful in pharmaceutical applications. This is why chitosan has been used as a safe sample in pharmaceutical formulations in the last 2 decades. The cationic properties of chitosan and the presence of a series of reducing functional groups have led to the use of this polymer in drug delivery systems with controlled release. Since chitosan has hydrophilic properties, it also increases the absorption of hydrophilic drugs. Chitosan as a coating has many advantages such as the controlled release of drug for a long time, the ability to carry more drug, and bioadhesive properties than uncoated particles. Drug release from the chitosan-based carrier depends on the morphology, size, density, and cross-linking of the particle system, as well as the chemical and physical properties of the drug, etc. In drug delivery systems, the solubility of chitosan can be reduced by cross-linking it with other substances. The release property of chitosan decreases with the increase in the concentration of the cross-linker [24–31].

Lewandowska-Łańcucka et al. in 2013 synthesized and investigated iron oxide nanoparticles modified with chitosan and silica coating. For the synthesis, they used a new and improved method, which was safe and cost-effective and did not require a template or surfactant and also did not require a high calcination temperature. The results showed that the final particles are spherical and 300 nm in size and have a magnetic saturation value of 22.1 emu/g. The properties of these composites showed that these particles can be used for engineering and medical applications [15]. In a research conducted by da Silva et al. in 2014, antibiotics were loaded into a nanostructure made of Fe_3_O_4_ nanoparticles with a titania–pectin shell and then slowly released using a remote magnetic field. By using this smart system in drug release in this research (use of magnetic field), there was a significant reduction in the amount of amoxicillin drug release. In other words, the drug levels were preserved in the acidic environment during the experiment. In fact, the presence of Fe_3_O_4_ nanoparticles played an important role in controlling the release compared to the use of pure pectin microspheres [11]. In 2014, Ding et al. synthesized iron oxide nanoparticles modified with the CM-B-CD polymer cross-linked with chitosan for the delivery of the anticancer drug of 5-fluorouracil. The results showed that the presence of free carboxymethyl groups around the holes increases the ability to load the carrier for insoluble anticancer drugs and the drug loading efficiency reached 97.6% [32]. In another research conducted in 2014 by Xiang et al., Fe_3_O_4_/chitosan/TiO_2_ nanocomposite was synthesized as a photocatalyst material. The research showed that the synthesized nanocomposites have good absorbent, photocatalytic, reduction, and magnetic properties and had potential applications in the absorption and degradation of organic pollutants in wastewater [33]. In a study conducted by Moazen and Panahi in 2017, the synthesis of magnetic iron oxide nanoparticles was achieved through a simple coprecipitation method and then modified by (3-mercaptopropyl)trimethoxysilane followed by grafting thermosensitive polymer *N*-isopropylacrylamide and biopolymer chitosan [34]. In a study conducted by Esmaili and Ghobadianpour in 2016, superparamagnetic Fe_3_O_4_ nanoparticles were synthesized by the coprecipitation method as the core and then covered by chitosan polymer and polyethylene glycol 400 as a stabilizer against the reticuloendothelial system. Reticuloendothelial was used, and at the end, vancomycin was loaded into the synthesized nanocomposite. The results showed that by using the slow release system of vancomycin drug that was used, it is possible to reduce the intervals of taking the drug by the patient, which reduces the side effects of the drug and, as a result, the satisfaction of the patient. It was also shown that by using this system, vancomycin can affect Gram-negative bacteria and destroy their cell walls [35]. In 2016, Samira Kariminia et al. synthesized iron oxide nanoparticles modified with chitosan as a carrier for the pH-sensitive drug Ciprofloxacin, and the drug loading efficiency of the nanocarrier drug was more than 99%. In this research, ultrasonic radiation as an external stimulus was used to release the drug. The results showed that the release of the drug was increased by a factor of 68.45 compared to not using ultrasonic [6]. In other works, iron oxide magnetic nanoparticles modified with starch/carboxymethyl cellulose polymer mixture were used as a carrier for the targeted delivery of the antiviral drug [36–38]. In 2019, Ayoub Moghadam synthesized MnFe_2_O_4_/chitosan nanocomposite to control the release of the ofloxacin drug, and the results showed that the drug loading efficiency of the nanocomposite was more than 88%. In vitro studies showed that the drug is released continuously for the first 7 hours, followed by a slow release over a controlled period of 3 days, where the release time of the drug is comparably very noticeable to the lifetime of the drug (between 8 and 9 h) [13]. The present study was conducted with the aim of synthesizing magnetic nanoparticles with chitosan and titania coating as a new nanocarrier for vancomycin drug (Figure 1(b)). In general, the main goal and innovation of this research is the synthesis of surface-modified magnetite nanoparticles with chitosan and the investigation of the effect of titania coating on the controlled release of vancomycin drug for these superparamagnetic nanoparticles.

## 2. Experimental

### 2.1. Materials

Ferric chloride hexahydrate (FeCl_3_.6H_2_O), ferric chloride tetrahydrate (FeCl_2_.4H_2_O), and chitosan (CS) polymer with low molecular weight were purchased from Sigma Aldrich. Acetic acid (CH_3_COOH), ammonium hydroxide (NH_4_OH), potassium dihydrogen phosphate (KH_2_PO4), disodium hydrogen phosphate (Na_2_HPO_4_), sodium hydroxide (NaOH), Tween 80 (80 Tween), and sodium chloride (NaCl) were prepared from Merck. Titania nanopowders, with particle sizes below 100 nm, were also obtained from Sigma Aldrich. All experiments were conducted using deionized water.

### 2.2. Synthesis of Nanocarrier, Drug Loading, and Coating of Titania

#### 2.2.1. Synthesis of Fe_3_O_4_ Nanoparticles

Co-precipitation chemical method was used for the synthesis of iron oxide magnetic nanoparticles. For this purpose, a 2:1 stoichiometric ratio of FeCl_2_.4H_2_O/FeCl_3_.6H_2_O was weighed and each of the iron salts was dissolved in 100 mL of deionized water by a magnetic stirrer. After complete dissolution, the resulting solutions were mixed inside a three-headed balloon; then, the final solution was stirred at a high speed under a continuous flow of argon gas at a temperature of 70°C. The presence of argon gas prevents oxidation. In the next step, a 3 M solution of sodium hydroxide was added to the stirring solution drop by drop. After adding alkaline sodium hydroxide solution, the color of the solution changed from dark orange to black immediately, and the pH of the solution was kept constant at 12. In order to achieve a uniform mixture and after adding the alkaline solution, the stirring of the solution was continued for 60 min at a temperature of 70°C. The isolated material was thoroughly washed several times with deionized water and alcohol to remove any remaining impurities, ensuring the pH was adjusted to a neutral value of 7. Finally, the collected nanoparticles were dried in an oven at 70°C for 24 h.

#### 2.2.2. Synthesis of Chitosan-Coated Magnetite Nanoparticles (Fe_3_O_4_@CS)

To make surface-modified magnetite nanoparticles with chitosan, first 100 mL solution (1%) of acetic acid was prepared. Then, 0.5 g of purchased chitosan powder was added to the previous solution and stirred by a magnetic stirrer for 30 min until chitosan was completely dissolved. 0.5 g of the obtained nanoparticle in the previous step was dispersed in 100 mL of deionized water and stirred by a magnetic stirrer for 30 min at high speed. The solution containing nanoparticles was added to the chitosan solution, and the final mixture was stirred for 60 min at room temperature by a magnetic stirrer until a brownish black suspension was formed. After finishing the coating process, a magnet was used to separate the coated magnetic nanoparticles and the unreacted chitosan was discarded. The coated magnetic nanoparticles underwent multiple washes with deionized water and were subsequently dried in an oven at a temperature of 50°C.

#### 2.2.3. Drug Loading Experiments

##### 2.2.3.1. Drug Loading and Investigation of the Effect of the Ratio of Vancomycin to Nanocarriers on Drug Loading Efficiency

Vancomycin was loaded on surface-modified magnetite nanoparticles with chitosan, which have amino groups, during the following steps, and the molar ratio of chitosan to nanoparticles was optimized to achieve the highest loading efficiency. For this purpose, a solution with a concentration of 1000 mg/L of vancomycin and deionized water was prepared. 0.3 g of Fe_3_O_4_@CS nanocarriers with different weight ratios according to Table 1 were dispersed in three balloons containing 50 mL of deionized water and stirred by a magnetic stirrer at high speed (according to Table 1, samples were named with codes A_1_, A_2_, and A_3_). Using a pipette, some of the drug solution was removed and added to the solution of nanoparticle coated with chitosan while stirring so that the ratio of vancomycin/Fe_3_O_4_@CS for all three balloons is equal to 1:10. Then, the resulting mixture was stirred at room temperature for 24 h by a magnetic stirrer to ensure proper interaction between vancomycin with negative charge and chitosan molecules with positive charge. Finally, the drug-loaded nanocarriers were separated by magnets. The amount of unabsorbed drug in the upper solution was measured using a UV–vis spectrometer at a wavelength of 280 nm. Then, using relationship 1, the drug loading efficiency on the nanocarrier was measured:

(1) $$\begin{array}{c} \%\text{LE}=\frac{\left(\text{total concentration of drug}-\text{concenteration of free drug}\right)}{\text{total concentration of drug}}\times 100. \end{array}$$

To calculate the amount of drug absorption, it is necessary to draw the calibration curve of absorption according to the drug concentration. For this purpose, first, 10 mg of vancomycin antibiotic was dissolved in 100 mL of distilled water to prepare the stock solution (100 ppm). Then, standard solutions in the concentration range of 10–100 ppm were prepared using the stock solution and their absorbance was calculated using a UV–Vis spectrometer at a maximum wavelength of 280 nm. This experiment was repeated three times, and the average absorption was calculated. Then, using the numbers obtained, the calibration curve of the vancomycin was drawn at a wavelength of 280 nm.

##### 2.2.3.2. Effects of pH and Contact Times on Drug Loading Efficiencies

To evaluate how pH and contact time affect drug loading efficiency, nanocarrier solutions with the ideal ratio (designated as A_3_) were formulated at pH values between 3 and 9 and stirred for various durations. The solutions were then subjected to centrifugation at 1000 rpm, and the drug loading efficiency was calculated using the specified formula (Eq. (1)).

#### 2.2.4. Coating of the Vancomycin-Loaded Nanocarriers With Titania

To put the titania NP coating on the vancomycin-loaded nanocarriers (Fe_3_O_4_@CS@vancomycin) obtained from the previous three steps, first 0.2 g of A_3_ nanocarrier (according to the results obtained from the drug loading analysis, this sample has the highest amount of drug loading) and the titania nanopowder were dispersed in 100 mL of water, separately, and then mixed together. Some Tween 80 was added to the solution as an emulsifier. Then, the resulting solution was stirred for 2 h by a magnetic stirrer. Finally, using a magnet, the final nanocarriers were settled and separated, washed with deionized water, and dried in an oven at 50°C.

### 2.3. Investigation of the Effect of Titania Coating on Vancomycin Release

To investigate the effect of titania coating on vancomycin release from the synthesized nanocarrier, two nanocarrier samples containing the same amount of drug before and after titania coating (for sample A_3_ with the maximum amount of drug loading) were placed separately under the same conditions in the pH values 5 and 7.4 of phosphate buffer solutions, which, respectively, corresponds to the pH of cancer cells and physiological pH of the body, at a temperature of 37°C on a low speed stirrer. Gradually, the loaded drug is released from the magnetic nanocarriers, and with the passage of time, the concentration of the drug inside the buffer increases. To measure the concentration of the released vancomycin, 2 mL of the buffer solution is separated at specific time intervals and its absorbance is read by a UV–Vis spectrophotometer at maximum wavelength of absorption at 280 nm against the calibration curve. At this stage, there is no need to use a dialysis bag because magnetic nanoparticles can be separated from the upper solution by a magnet, and this is an advantage of using magnetic nanoparticles. After each sampling, 2 mL of fresh buffer was added to the solution containing nanocarriers to release the drug from the nanocarriers in the same volume of the solution. The % cumulative release of drug in different time periods was calculated according to equation (2):

(2) $$\begin{array}{c} \text{Cumulative release}\left(\%\right)=\frac{\text{mg}\left(\text{drug released}\right)}{\text{mg}\left(\text{total drug}\right)}\times 100. \end{array}$$

In this context, mg (drug released) denotes the amount of drug released at time *t*, while mg (total drug) indicates the total amount of drug encapsulated within the drug-loaded magnetic nanoparticles [13].

### 2.4. Kinetics of Drug Release

Data of drug release from the nanocarrier without/with titania coating were analyzed with different kinetics models, such as, zero order, Korsmeyer–Peppas, first order, and Higuchi kinetics. The model with the highest correlation coefficient (*R*^2^) was chosen to represent the drug release behavior from the nanocarrier. The linear equations corresponding to these models are as follows:

• Higuchi kinetics [16, 19]:
  (3) $$\begin{array}{c} Q_{t}={Kt}^{1/2}. \end{array}$$
• First-order kinetics [36]:
  (4) $$\begin{array}{c} \ln\left(1-Q_{t}\right)=K_{t}. \end{array}$$
• Korsmeyer–Peppas kinetic [14]:
  (5) $$\begin{array}{c} \frac{Q_{t}}{Q_{\infty }}={Kt}^{n}. \end{array}$$

Here, *Q*_*∞*_ and *Q*_*t*_ represent the cumulative concentration of the released drug at infinite time and at time ttt, respectively. K is the release rate constant, and *n* is the release coefficient, which characterizes the type of diffusion. If *n* < 0.5, the diffusion mechanism follows Fickian behavior, indicating that the drug diffuses at a slower rate than the polymer relaxes. If *n* = 1, polymer relaxation governs the water penetration into the carrier, known as Case II transport. Values of *n* between 0.5 and 1 indicate a non-Fickian or anomalous diffusion mechanism, where both polymer relaxation and diffusion control the water penetration into the carrier [39–41].

### 2.5. In Vitro Cytotoxicity

Due to the high importance of the nanocarrier toxicity for drug delivery, the cytotoxic effects of pure vancomycin, Fe_3_O_4_@CS nanocarrier, and vancomycin-loaded nanocarrier without/with titania coating were investigated using the MTT test. HeLa cells were plated in 96-well plates and kept in a culture medium for 24 h. Then, the cells were incubated with nanocarriers at different concentrations (100–500 μg/mL).

To calculate the ability of free vancomycin, Fe_3_O_4_@CS@TiO_2_ NP nanocarrier, and vancomycin -loaded nanocarrier to kill HeLa cells, the cytotoxicity of these samples at different concentrations was calculated so that the concentration of vancomycin was similar (100–500 μg/mL). Following a 24-h incubation period, MTT solution was introduced to each well and subsequently incubated in a humid environment containing 95% air and 5% CO_2_ for 4 h at a temperature of 37°C. The supernatant containing MTT was then replaced with 100 μL of dimethyl sulfoxide to dissolve the formazan crystals, followed by an additional 30-min incubation. Finally, the absorbance of the samples was recorded at 570 nm, with background subtraction performed at 630 nm.

### 2.6. Characterization

The IR spectrum of the samples was drawn using an FTIR spectrophotometer (Thermo Company, AVOAR model) in the wavenumber range of 400–4000 cm^−1^. The diffraction pattern of the samples was drawn to determine the crystal structure and phase identification by X-ray diffraction instrument (PHILIPS Company, pw1730 model). A scanning electron microscope (FESEM, TESCAN MIRA3 model) and transmission electron microscope (TEM, FEI 120 KV Tecnai G2 Sprint BioTwin) were used to observe the morphology and determine the size of nanocarriers. A SQUID magnetometer (Quantum Design MPMS-XL-7) was used to investigate the magnetic properties of synthesized nanoparticles and nanocarriers. The amount of drug absorption and release was calculated using a UV–Vis spectrometer (Agilent 8453). The specific surface area and pore size distribution of samples were determined from N_2_ adsorption–desorption measurements (BEL Japan).

## 3. Results and Discussion

### 3.1. Characterization of Loaded and Unloaded Nanocarriers Without/With Titania Coating

#### 3.1.1. XRD

**Figure 2:**
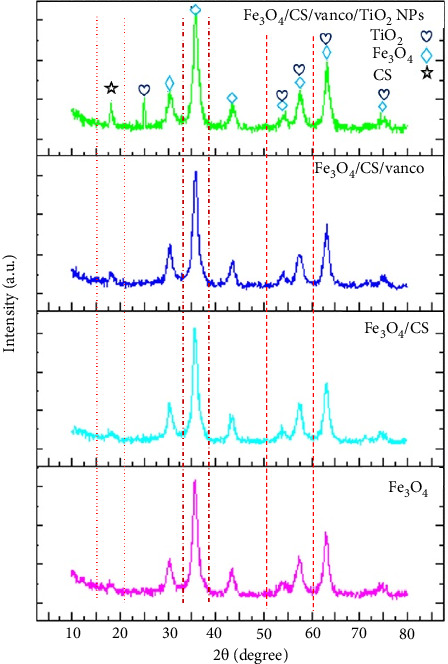
XRD diffraction patterns of Fe_3_O_4_, Fe_3_O_4_@CS, Fe_3_O_4_@CS@vanco, and Fe_3_O_4_@CS@vanco@TiO_2_ samples.

X-ray diffraction patterns of Fe_3_O_4_, Fe_3_O_4_@CS, Fe_3_O_4_@CS@Vanco, and Fe_3_O_4_@CS@Vanco@TiO_2_ NP samples are shown in Figure 2. Magnetite nanoparticles have six distinct peaks corresponding to planes (220), (311), (400), (422), (511), and (440), which have been determined at 2*θ* angles of 30, 35, 43, 53, 57°, and 63°, respectively. By comparing this spectrum with the magnetite card (01-075-0033) and using the Xpert software, it is clear that all the peaks are consistent with the magnetite peak and no side phase is observed. The obtained results show that the crystal structure of magnetite nanoparticles is consistent with the structure of inverted cubic spinel [6, 41]. The characteristic peak angles of magnetite nanoparticles remained unchanged after surface modification with chitosan polymer and drug, which indicates the preservation of the crystalline structure of nanoparticles with chitosan and drug loading. The loading of chitosan and the drug vancomycin has only led to a change in the intensity of the peaks, which indicates the presence of a new compound in the structure and shows that the act of coating with chitosan and loading the drug has been done successfully. Vancomycin drug has no characteristic peak, and the broad peak observed at 2*θ* = 20° corresponds to chitosan. As shown, in the vancomycin-loaded nanocarrier and coated with titania nanoparticles, in addition to the characteristic peaks of magnetite, the main peak with the highest intensity of anatase at 2*θ* = 25° is clearly identified and confirms the presence of titania coating on the nanocarrier. Other characteristic peaks related to the anatase phase of titania occur at angles of 54, 56, and 63, which overlap with magnetite peaks [6, 33, 42].

#### 3.1.2. FTIR

**Figure 3:**
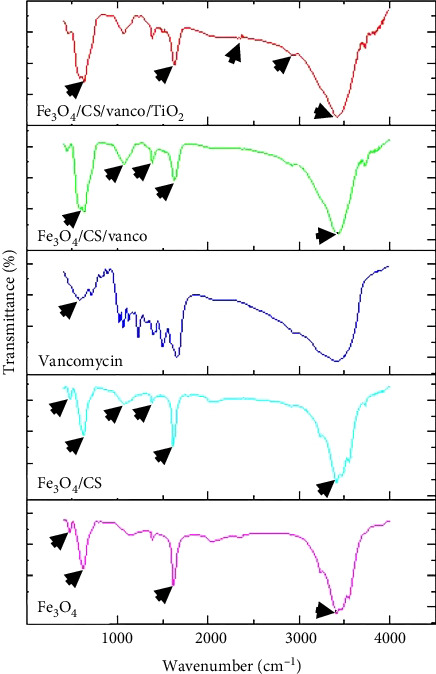
FTIR spectra of Fe_3_O_4_, Fe_3_O_4_@CS, vancomycin, Fe_3_O_4_@CS@vanco, and Fe_3_O_4_@CS@vanco@TiO_2_ NP samples.

In order to show the formation of magnetite nanoparticles and their loading with chitosan, vancomycin, and titania, FT-IR analysis of Fe_3_O_4_, Fe_3_O_4_@CS, vancomycin, Fe_3_O_4_@CS@Vanco, and Fe_3_O_4_@CS@Vanco@TiO_2_ NP samples in the wavelength range of 400–3900 cm^−1^ were done and the corresponding spectra of each sample are given in Figure 3. The broad absorption band in the range of 3400–3500 cm^−1^ and the absorption peak in the range of 1614 cm^−1^ in all samples are related to the stretching oscillation of the O–H bond of water absorbed on the surface of the nanocarriers, and water in the environment, respectively. Also, the absorption bands observed in the spectrum of magnetite NPs at 477 and 624 cm^−1^ are related to the vibration of the Fe–O bond. The presence of these peaks in the mentioned areas confirms the formation of iron oxide structure [12, 43]. It can be seen that by the coating of magnetite nanoparticles with chitosan, the location of these peaks slightly shifted to 479 and 627 cm^−1^, which indicates the interaction between chitosan and magnetite NPs.

In the FTIR spectrum of the Fe_3_O_4_@CS sample, the peaks observed at 1079, 1386, and 1619 cm^−1^ correspond to the stretching vibration of C–O–C, the bending vibration of O–H, and the stretching vibration of N–H, respectively, within the chitosan structure. The presence of these peaks indicates that the magnetite nanoparticles are effectively coated with chitosan [44, 45].

In the diagram of Fe_3_O_4_@CS@Vanco, the stretching vibration observed at 1250 cm^−1^ and the vibrations observed at 1630 cm^−1^ and 1504 cm^−1^ correspond to the phenol ring, the C = O bond vibration, and the C = C vibration, respectively. The peak formed at 3417 cm^−1^ is related to the phenol ring with hydrogen bond in the vancomycin molecule. Also, the slight change in the position of the Fe–O bond oscillation and the shift to the wavelengths of 485 cm^−1^ and 634 cm^−1^ show that vancomycin is well loaded in the chitosan polymer.

The peak observed at a wavelength of 648 cm^−1^ in the spectrum of Fe_3_O_4_@CS@Vanco@TiO_2_ NPs is related to the Ti–O–Ti bond, which partially overlaps with the Fe–O bond peak in magnetite. The observed peaks at 2900 and 2350 cm^−1^ are related to the vibration of CH_2_ and CO_2_ groups in the ambient atmosphere, respectively. Also, the observed peaks at 1638 and 3400 cm^−1^ are related to the hydroxyl group adsorbed on the nanocarrier. By comparing the obtained spectra with the reference spectra, it can be concluded that all components are loaded in the Fe_3_O_4_@CS@Vanco@TiO_2_ NP sample [6, 33, 46, 47].

#### 3.1.3. VSM Analysis

**Figure 4:**
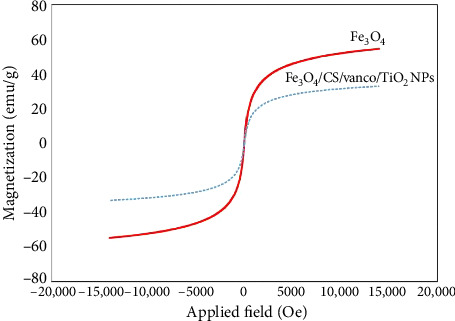
VSM spectrum of Fe_3_O_4_ MNPs and the final nanocarrier.

Since in this research, the reason for using magnetic nanoparticles is to target the carrier using an external magnetic field, therefore, it is very important and necessary to determine the magnetic properties of these nanoparticles. The VSM curve of magnetite nanoparticles and the final nanocarrier is shown in Figure 4. Since the hysteresis and magnetic inhibition are not observed in the diagram, it can be concluded that the nanoparticle is superparamagnetic. VSM curves show the property of superparamagnetism or ferromagnetism of the material. Superparamagnetism is one of the necessary properties for targeted magnetic carriers, and one of the criteria for identifying a product as superparamagnetism is the absence of hysteresis loop [48, 49]. The saturation magnetization of magnetite nanoparticles and the final nanocarrier is 54.73 emu/g and 33.05 emu/g, respectively, where a decrease of the saturation magnetization is due to the non-magnetic coatings formed on the iron oxide nanoparticles. However, the amount of saturation magnetization is sufficient for the intended purpose.

#### 3.1.4. FESEM and TEM

**Figure 5:**
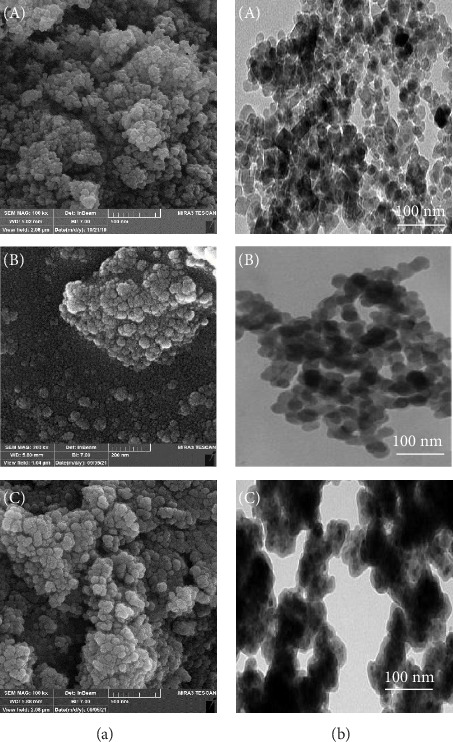
FESEM (a) and TEM (b) images of (A) Fe_3_O_4_ nanoparticles, (B) Fe_3_O_4_@CS@vanco, and (C) Fe_3_O_4_@CS@vanco@TiO_2_ samples.

Figure 5 shows the FESEM (left) and TEM (right) images of (a) magnetite nanoparticle, (b) magnetite nanoparticle modified with chitosan, and (c) the final nanocarrier. Panel a shows that magnetite nanoparticles are strongly agglomerated and clustered, which is due to their high specific surface and surface free energy, as well as their dipole–dipole magnetic force. The average size of magnetite nanoparticles is about 20 nm. By surface modification of nanoparticles with chitosan, drug loading, and subsequent coating with titania, the size of the nanocarrier has increased compared to the original magnetite nanoparticles and the amount of agglomeration has decreased. The TEM images clearly show the loading of chitosan and titania on magnetite nanoparticles. Applying a coating on the surface of nanoparticles will reduce the specific surface area and surface energy of nanoparticles, as well as reduce the magnetic forces between magnetite nanoparticles, which prevents of sticking of particles together and leads to a decrease in the amount of agglomeration, These results are consistent with the results of the VSM analysis (Figure 4).

#### 3.1.5. Zeta Potential Measurements

**Figure 6:**
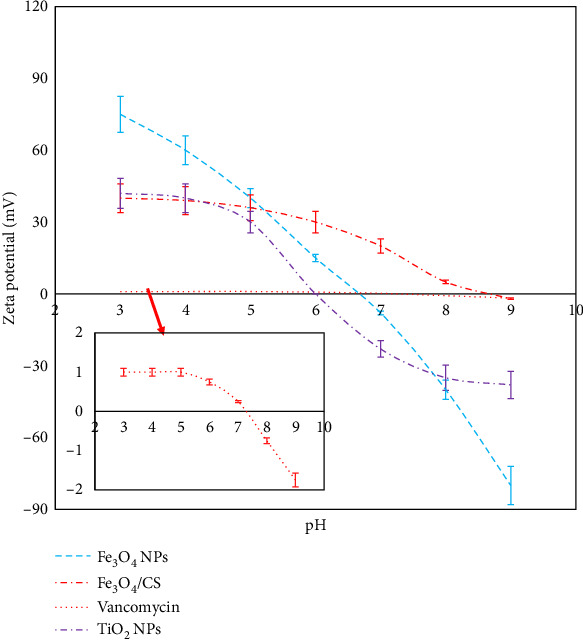
Zeta potential changes with pH for Fe_3_O_4_ MNPs, Fe_3_O_4_@CS nanocarrier, vancomycin drug, and TiO_2_ NPs.

To confirm the pH responsivity on net charge and determine the appropriate pH for drug loading, the zeta potentials of Fe_3_O_4_ NPs, Fe_3_O_4_ NPs/CS nanocarrier, vancomycin, and TiO_2_ NPs were determined at different pH values. As shown in Figure 6 (in order to better show the changes, the curve of vancomycin zeta potential changes vs. pH is shown again in the inset), the zeta potential–pH trends of the Fe_3_O_4_/chitosan nanocarrier were clearly dominated by the chitosan polymeric shell. This is likely due to the efficient polymeric coating of Fe_3_O_4_, resulting in core/shell nanoparticles. From an electrokinetic perspective, these core/shell NPs exhibit characteristics that are qualitatively similar to chitosan [50, 51].

The zeta potential decreased from 40.0 to 0 as the pH increased up to 8.5, demonstrating that the nanocarrier exhibits good pH responsiveness. Due to the positive charge on the nanocarriers, electrostatic interactions occur with the negatively charged vancomycin and TiO_2_ nanoparticles. As a result, a pH of 7.4 was selected as the optimal condition for drug loading.

#### 3.1.6. BET–BJH Analysis

**Figure 7:**
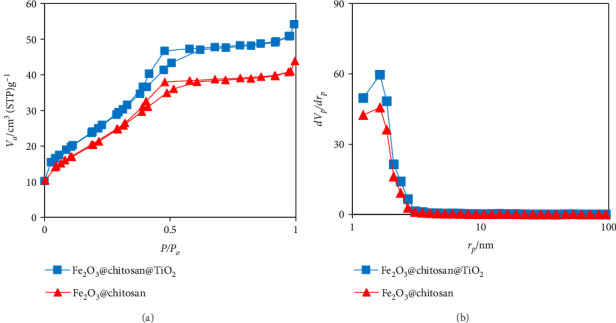
(a) The adsorption–desorption isotherm and (b) pore size distribution curve of samples.

The BET–BJH analysis (Figure 7) was employed to ascertain the surface area and pore size of the Fe_3_O_4_/chitosan and Fe_3_O_4_/chitosan/TiO_2_ samples. The specific surface area values for the Fe_3_O_4_/chitosan and Fe_3_O_4_/chitosan/TiO_2_ samples were measured at 74 and 86 m^2^/g, respectively. According to the BET analysis (see Figure 7(a)), the specific surface area exhibited to be higher for Fe_3_O_4_/chitosan/TiO_2_ compared to that for Fe_3_O_4_/chitosan due to the presence of TiO_2_ NPs in the nanocarrier composition. The physisorption isotherm of the samples exhibited a type IV classification, characterized by a hysteresis loop of type H2. An H2 hysteresis loop characterizes a mesoporous material composed of spherical particles, which exhibit a relatively wide distribution of pore sizes and a pore morphology frequently described as resembling an “ink bottle” [52]. The pore size distribution illustrated in Figure 7(b), derived from the BJH analysis, is intended to assess the quantity and dimensions of pores within the samples. In this context, the curve for Fe_3_O_4_/chitosan/TiO_2_ indicates an increase in the number of pores attributed to the presence of TiO_2_ NPs. The mean pore sizes were 1.65, and 1.67 nm for Fe_3_O_4_/chitosan and Fe_3_O_4_/chitosan/TiO_2_ samples, respectively.

### 3.2. Vancomycin Loading Studies in the Nanocarrier

#### 3.2.1. Effect of Different Ratios of CS to Fe_3_O_4_ on Drug Loading Efficiency

**Figure 8:**
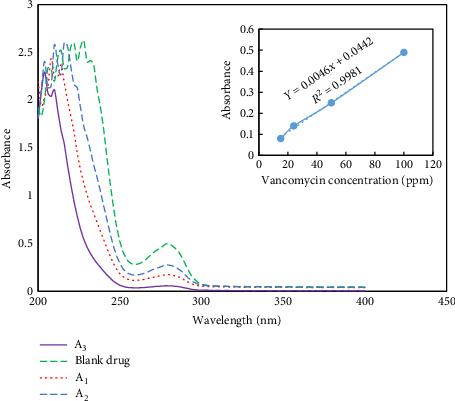
UV–Vis absorption spectrum of A_1_, A_2_, and A_3_ samples and vancomycin drug.

Figure 8 (inset shows the calibration curve of vancomycin) shows the UV–Vis spectrum of drug absorption in Fe_3_O_4_@Cs nanocarriers with different ratios of chitosan to magnetite nanoparticles, and the amount of drug absorption has been calculated by changing the molar ratio for samples A_1_, A_2,_ and A_3_ in Table 2. As is clear, as the ratio of chitosan polymer to iron oxide nanoparticle increases, the drug loading percentage also increases, which can be due to the increase of amine groups on the surface of chitosan with a positive charge for the absorption of drug, which is negatively charged. For this purpose, we chose the best formulation in which the ratio of magnetite nanoparticle/polymer is 2:1 (sample A_3_) and has the highest amount of drug loading to perform the next steps of the work and coating of the titania.

#### 3.2.2. Impact of Different Nanocarrier to Drug Ratios on the Efficiency of Drug Loading

**Figure 9:**
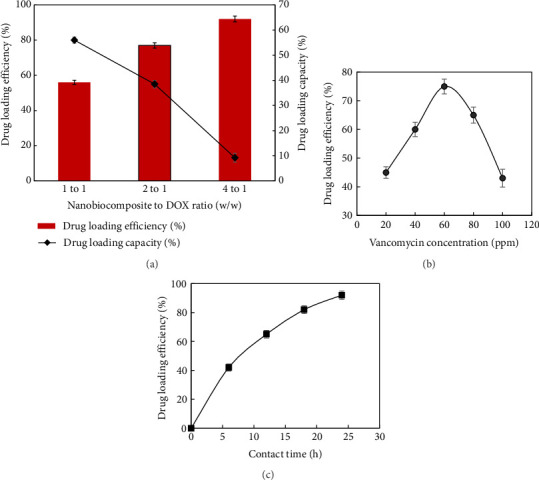
(a) Effects of different nanocarrier to vancomycin ratios on loading efficiency and loading capacity, and the effect of (b) concentration of vancomycin and (c) contact time on drug loading efficiency.

To evaluate the highest drug loading efficiency, different drug-to-nanocarrier ratios (sample A_3_) were examined. Various amounts of the nanocomposites were combined with 10 mL of the drug solution, using nanocarrier-to-vancomycin ratios of 1:1, 2:1, and 4:1. The drug loading efficiencies and capacities for these ratios are illustrated in Figure 9(a). The highest drug loading efficiency, 92%, was achieved at a nanocarrier to drug ratio of 4:1. In contrast, the maximum drug loading capacity, around 80%, occurred at a 1:1 ratio.

#### 3.2.3. Impact of pH on Efficiency of Drug Loading

The impact of the pH of the nanocomposite solution, ranging from 3 to 9, on drug loading efficiency was examined, and the results are presented in Figure 9(b). As can be seen in this figure, changing the pH of the nanocomposite solution has a remarkable effect on the amount of vancomycin loaded. Chitosan possesses primary amino groups with a pK_a_ of approximately 6.3. At pH levels below this value, the amino groups are predominantly protonated, rendering chitosan a water-soluble cationic polyelectrolyte. In contrast, when the pH increases above the pK_a_, these amino groups deprotonate, leading to a loss of solubility in the polymer [53]. In contrast, vancomycin exhibits six distinct pK_a_ values: 7.75 and 8.89 (basic), as well as 2.18, 9.59, 10.4, and 12.0 (acidic). The specific functional groups contributing to these pK_a_ values are illustrated in Figure 1(b). Also, the net positive charge of the vancomycin decreases with increasing pH up to 8.5 and starts to increase from this value onwards. Calculations were made according to the following equations: net charge = 10^–pH^/(10^–pKa^ + 10^–pH^) for bases and −10^–pKa^/(10^–pKa^ + 10^–pH^) for acids [54]. Therefore, the results of the pH dependence and its effect on cross-linking of drug with chitosan could be explained by the charge distribution of chitosan and vancomycin. The higher drug loading efficiency at pH close to 6 (92%) was due to the maximized electrostatic interaction between the protonated chitosan amino groups and the dissociated carboxyl groups of vancomycin. At pH values below 5, drug loading efficiency decreases due to charge–charge repulsion, while at pH values above 7, the reduction in the number of positively charged amino groups of chitosan leads to lower efficiency. These results further confirm that drug loading efficiency is primarily governed by the electrostatic interactions between the nanocarrier and vancomycin.

#### 3.2.4. Effect of Contact Times on Efficiency of Drug Loading

In order to investigate the effect of contact time between the drug and nanocarrier on drug loading efficiency, the amount of drug loading inside the nanocarrier was investigated at different contact times, and the results are shown in Figure 9(c). The results showed that with the increase of the contact time, the drug loading efficiency increases, so that the drug loading efficiency reaches about 90% at a contact time of 25 h.

### 3.3. Vancomycin Release Behavior

**Figure 10:**
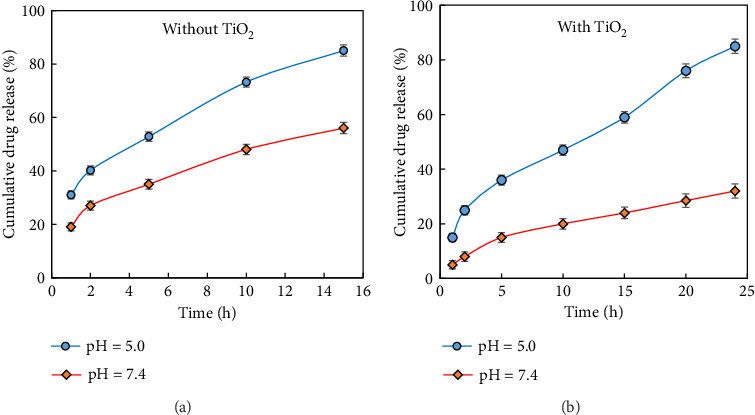
Drug release profiles of nanocarrier (a) without TiO_2_ coating and (b) with TiO_2_ coating at pH 5 and 7.4 at 37°C.

We investigated the release behavior of the nanocarriers with/without titania coating at two solutions with different pH (5 and 7.4) at 37°C as shown in Figure 10. The release of the drug from the nanocarrier is done due to the penetration of water into the polymer matrix and the subsequent penetration of the drug into the aqueous medium. The release behavior of nanocarrier is affected by pH and TiO_2_ coating as shown in Table 3. It is clear that the swelling of the nanocarrier increases with increased pH, which may be caused by hydrogen-bonding electrostatic and interaction. This behavior is particularly linked to the pH-sensitive groups within the polymer matrix, which respond to changes in pH, leading to swelling [55, 56].

The release of vancomycin from the nanocarrier is driven by the diffusion of water into the polymer network, followed by the penetration of the drug into the aqueous medium. Consequently, the release of the drug is influenced by the swelling behavior of the polymer within the nanocarrier. At pH 5, the drug release is pH-dependent due to the restricted swelling of the polymer network. At pH 7.4, however, the nanocarrier demonstrates increased swelling due to the presence of hydrophilic groups like –OH and –COOH, which exhibit a stronger affinity for water. At this higher pH, the carboxylic acid groups ionize more efficiently, generating repulsive forces that cause the network structure to expand, thereby promoting greater swelling [56]. Also, as seen in Table 3, the swelling degree of the nanocarrier increases with titania coating noticeably. The results showed that by creating a titania coating, it causes interaction between groups on the polymers and creates a network with high cross-linking [57]. To sum up, the swelling behavior is significantly influenced by the content of added nanocarrier materials, even though it is a macroscopic effect that results from the overall combined contribution of nanostructures of the chemically modified network of the polymer matrix. This result can be due to the absorption properties of the obtained nanocarrier and the presence of titania nanoparticles, which causes a significant increase in the specific surface area [58]. Therefore, as shown in Figure 9, with the applied titania coating at higher pH, the swelling rate increases, and as a result, due to the hardening of water penetration in the polymer network, the cumulative drug release rate was greatly reduced.

### 3.4. Drug Release Kinetics

**Figure 11:**
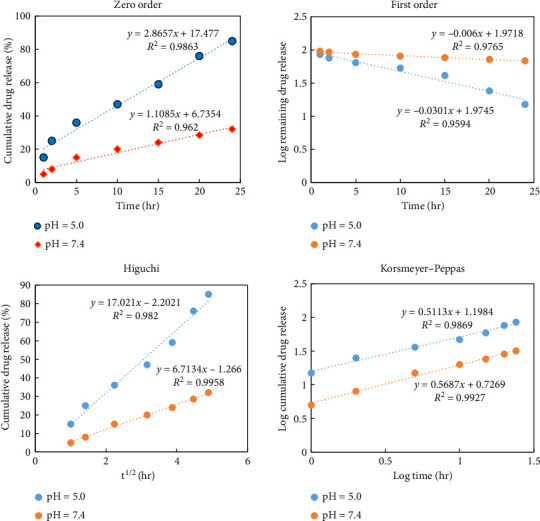
Vancomycin release kinetics of nanocarrier with TiO_2_ coating in solutions with pH 5 and 7.4 at 37°C.

The results obtained from modeling the release data are presented in Figure 11. By investigating the correlation coefficients (*r*^2^) of models in both basic and acidic conditions, it was approximated that the drug release kinetics is most fitted with the Higuchi and Korsmeyer–Peppas models.

The drug release rate is influenced by two concurrent processes: (1) water penetration into the system and (2) drug penetration, which is dependent on the extent of network swelling [59–61]. According to the linear plots of the Korsmeyer–Peppas model, the release exponent “*n*” was found to be 0.387 at pH 5, indicating that the drug release mechanism follows Fickian diffusion. At pH 7.4, however, “*n*” was 0.755, suggesting non-Fickian transport. At pH 7, the ionization of the –OH and –COOH groups in the nanocarrier generates negatively charged ions. As a result, repulsive forces between these ions trigger polymer relaxation, leading to drug release being governed by polymer relaxation and the penetration of both water and the drug into the release med [62].

### 3.5. In Vitro Cytotoxicity

**Figure 12:**
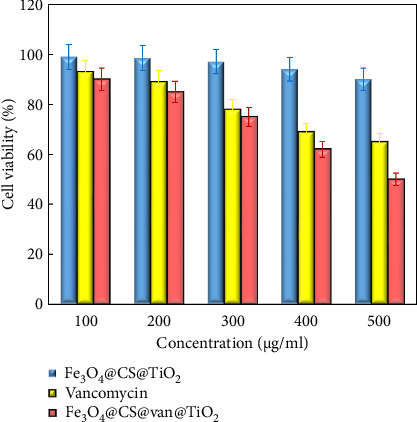
Cytotoxic effect of the Fe_3_O_4_@CS@TiO_2_ NPs, free vancomycin, and Fe_3_O_4_@CS@ Fe_3_O_4_@CS@ vanco@TiO_2_ NPs on HeLa cell viability was examined by MTT assay.

To assess the biocompatibility of the Fe_3_O_4_@CS@Titania nanocarrier, its in vitro anticancer effect was evaluated on HeLa cells using the MTT assay. As shown in Figure 12, prior to vancomycin loading, the nanocarrier displayed minimal cytotoxicity toward HeLa cells. At a higher concentration of 500 μg/mL, cell viability remained around 90%, demonstrating the good biocompatibility of the nanocarrier for drug delivery. After drug loading, the toxicity effect of vancomycin-loaded Fe_3_O_4_@CS@Titania nanocarrier depends on concentration. Cell viability reduced from 90 ±3% to 50 ±2% for a concentration of 100 μg/mL and 500 μg/mL, respectively. This should be because of the strong cellular uptake of vancomycin -loaded nanocarrier by HeLa cells and the toxic effect of released drug [63, 64]. Furthermore, the results display that using TiO_2_ NPs as a coating of nanocarrier not only increases the cellular internalization but also ensures the targeted drug delivery of vancomycin into the cells.

### 3.6. Conclusion

In this research, Fe_3_O_4_/chitosan/TiO_2_ NP novel nanocarrier was synthesized. Applying the titania coating reduces the magnetic property of the nanocarrier, but this value is sufficient for the use of the nanocarrier as a targeted delivery system. The loading efficiency for the sample with the optimal ratio was about 90.45%. The in vitro study of the drug release behavior showed that the implementation of a titania coating significantly diminishes the drug release rate. Specifically, approximately 90 ± 0.2% of the drug is released over a period of 16 h for samples without the titania coating, while samples with the coating exhibit a release time of 25 h. The MTT assay indicates that the application of TiO_2_ nanoparticles on the nanocarrier resulted in a decrease in cell viability from 90 ± 3% to 50 ± 2% at concentrations of 100 μg/mL and 500 μg/mL, respectively. The results show that the newly synthesized nanocarrier has great potential to be used as a targeted drug release system.

## Figures and Tables

**Table 1:** Coding of samples based on the weight ratio of chitosan to Fe_3_O_4_.

Sample code	Weight ratio	CS weight (gr)	Fe_3_O_4_ weight (gr)
A_1_	1: 1	0.5	0.5
A_2_	1: 2	0.25	0.5
A_3_	2: 1	1	0.5

**Table 2:** Drug absorption data in A_1_, A_2_, A_3_ nanocarriers.

Nanocarrier	Drug concentration of top solution (ppm)	Absorption percentage (%)
A_1_	19.74 ± 0.05	80.26
A_2_	28.21 ± 0.04	71.79
A_3_	9.50 ± 0.02	90.45

**Table 3:** The swelling data for nanocarriers without/with coating of TiO_2_ at pH 5 and 7.4.

Time (hr)	Swelling degree (without titania coating)		Swelling degree (with titania coating)	
	pH 5	pH 7.4	pH 5	pH 7.4
0	0	0	0	0
2	6.5 ± 0.01	20 ± 0.05	25 ± 0.04	52 ± 0.07
5	7 ± 0.03	33 ± 0.01	37 ± 0.07	72 ± 0.13
10	7.5 ± 0.02	44 ± 0.02	47 ± 0.09	74 ± 0.15
24	9 ± 0.04	47 ± 0.12	50 ± 0.02	75 ± 0.07
48	11 ± 0.07	51 ± 0.08	54 ± 0.11	77 ± 0.12

## Data Availability

Research data are not shared.
